# Wild Soybean Oxalyl-CoA Synthetase Degrades Oxalate and Affects the Tolerance to Cadmium and Aluminum Stresses

**DOI:** 10.3390/ijms21228869

**Published:** 2020-11-23

**Authors:** Peiqi Xian, Zhandong Cai, Yanbo Cheng, Rongbin Lin, Tengxiang Lian, Qibin Ma, Hai Nian

**Affiliations:** 1 The State Key Laboratory for Conservation and Utilization of Subtropical Agro-Bioresources, South China Agricultural University, Guangzhou 510642, China; pqxian@stu.scau.edu.cn (P.X.); zdcai@stu.scau.edu.cn (Z.C.); ybcheng@scau.edu.cn (Y.C.); linrbl@163.com (R.L.); liantx@scau.edu.cn (T.L.); maqibin@scau.edu.cn (Q.M.); 2 The Key Laboratory of Plant Molecular Breeding of Guangdong Province, College of Agriculture, South China Agricultural University, Guangzhou 510642, China; 3 The Guangdong Subcenter of the National Center for Soybean Improvement, College of Agriculture, South China Agricultural University, Guangzhou 510642, China; 4 Guangdong Provincial Laboratory of Lingnan Modern Agricultural Science and Technology, South China Agricultural University, Guangzhou 510642, China

**Keywords:** acyl activating enzyme 3, oxalyl-CoA synthetase, oxalate accumulation, Cd and Al tolerance, wild soybean

## Abstract

Acyl activating enzyme 3 (AAE3) was identified as being involved in the acetylation pathway of oxalate degradation, which regulates the responses to biotic and abiotic stresses in various higher plants. Here, we investigated the role of *Glycine soja* *AAE3* (*GsAAE3*) in Cadmium (Cd) and Aluminum (Al) tolerances. The recombinant GsAAE3 protein showed high activity toward oxalate, with a *K_m_* of 105.10 ± 12.30 μM and *V_max_* of 12.64 ± 0.34 μmol min^−1^ mg^−1^ protein, suggesting that it functions as an oxalyl–CoA synthetase. The expression of a GsAAE3–green fluorescent protein (GFP) fusion protein in tobacco leaves did not reveal a specific subcellular localization pattern of *GsAAE3*. An analysis of the *GsAAE3* expression pattern revealed an increase in *GsAAE3* expression in response to Cd and Al stresses, and it is mainly expressed in root tips. Furthermore, oxalate accumulation induced by Cd and Al contributes to the inhibition of root growth in wild soybean. Importantly, *GsAAE3* overexpression increases Cd and Al tolerances in *A. thaliana* and soybean hairy roots, which is associated with a decrease in oxalate accumulation. Taken together, our data provide evidence that the *GsAAE3*-encoded protein plays an important role in coping with Cd and Al stresses.

## 1. Introduction

Oxalic acid, the simplest dicarboxylic acid, occurs as a natural product in a wide range of plants, animals, microorganisms, rocks, and soil [1]. It is a common component of organisms and primarily accumulates as soluble oxalate, insoluble calcium oxalate, or a combination of these two forms [2]. The functional of oxalate in organisms depends on its chemical form and distribution [3,4].

In plants, oxalic acid plays important roles in the responses to both biotic and abiotic stresses. For example, studies have shown that there is a suppressive effect of oxalic acid on the vital movements of *Meloidogyne incognita* in tomato roots [5]. In *Sida rhombifolia*, calcium oxalate crystals improve the defense against herbivores [6]. Compared to biotic stress, many studies have focused on the role of oxalate in the response to abiotic stress, and it is perceived to play certain roles in calcium regulation, ion homeostasis, metal tolerance, and other pathways [7]. In terms of metal tolerance, two main mechanisms for the utilization of oxalate have been reported: exclusion and internal mechanisms [3]. The exclusion mechanism involves the excretion of oxalate into the environment by the roots and occurs in response to the stress caused by metal ions [8]. For instance, as shown in the study by Zhu et al., Cd-induced oxalate secretion from the tomato root apex contributes to preventing Cd from entering the roots [9]. In buckwheat, Zheng et al. reported a role for Al-induced oxalic acid secretion in Al resistance [8]. Furthermore, Yang et al. indicated that tolerant rice varieties reduced the Pb uptake of roots by increasing the synthesis and secretion of oxalate [10]. The internal mechanism involves the sequestration of noxious metal ions in the form of a nonphytotoxic oxalate complex within the non-sensitive portion of the plant [11]. According to Wang et al., high levels of oxalate accumulation induced by Cd stress in the roots of Chinese cabbage improve its internal tolerance to long-term Cd stress [12]. In addition, the Al-induced accumulation of oxalate in the roots reduces Al toxicity in buckwheat [11,13]. Most types of metal stress, such as Cd and Al stress, induce the accumulation of oxalate in plants [14,15]. However, due to the strong acidity of oxalic acid and because it is also considered a plant virulence factor [1], the excessive accumulation of oxalate would affect the normal metabolism and natural growth of plants, particularly in plants that do not normally accumulate oxalate. To date, Caryophyllaceae, Chenopodiaceae, and Polygonaceae are the main plant families characterized as oxalate accumulators [2], while the Leguminosae, such as wild soybean varieties, which are thought to be oxalate-non accumulators, are more likely to be poisoned by the accumulation of oxalate following metal stress. As shown in recent studies, the accumulation of oxalate and formate contribute to Al-induced inhibition of root growth in rice bean [14,16]. Additionally, in the study by Nakata et al., the overexpression of an oxalate synthesis gene in *Burkholderia mallei* increased the oxalate content in *A. thaliana*, resulting in a narrower leaf blade and a smaller rosette [17]. As mentioned in the study by Lou et al., the accumulation of oxalate is an early event that arrests the growth of plant roots under Al stress [14]. Therefore, oxalate metabolism must be controlled in plants.

Three different pathways for the degradation of excess oxalate have been identified in plants: oxidation, decarboxylation, and acetylation. Oxalate was oxidized to CO_2_ and H_2_O_2_ under the action of oxalate oxidase, while oxalate decarboxylase directly catalyzes the degradation of oxalate into formic acid and CO_2_ [18]. The third acetylation pathway is jointly mediated by four enzymes: oxalyl-CoA synthase, oxalyl-CoA decarboxylase, formyl-CoA hydrolase, and formate dehydrogenase. Oxalic acid is transformed into oxalyl-CoA by oxalyl-CoA synthetase and then degraded into formyl-CoA and CO_2_ through the actions of oxalyl-CoA decarboxylase. Next, formyl-CoA hydrolase catalyzes the degradation of formyl-CoA to form formate, and finally, CO_2_ is produced by formate dehydrogenase [19]. To date, oxalyl-CoA synthetase, the enzyme that catalyzes the first step in the CoA-dependent pathway, has been identified in *A. thaliana* as a protein encoded by *ACYL-ACTIVATING ENZYME 3* (*AAE3*) [20]. In addition, Yang et al. revealed that oxalyl-CoA decarboxylase (*ZmOCD1*) degrades oxalyl-CoA in maize [19]. Moreover, formate dehydrogenases (FDHs) have been generally regarded as NAD-dependent enzymes that catalyze the oxidation of formate to CO_2_ [21]. However, the gene encoding the formyl-CoA hydrolase remains unknown. Among the acetylation degradation pathways, oxalyl-CoA synthetase has consistently been shown to be involved in many responses of biotic and abiotic stresses. For example, Foster et al. reported a role for *Medicago truncatula AAE3* (*MtAAE3*) in regulating the accumulation of calcium oxalate and defending against phytopathogens that secrete oxalate [22]. According to Lee et al., the *CaSIG4* encoding a putative acyl-CoA synthetase plays a role in signaling in plant defense [23]. Lou et al. postulated that *VuAAE3* (*Vigna umbellata AAE3*) plays a critical role in Al tolerance mechanisms [14]. Furthermore, Liu et al. reported a negative regulatory effect of *OsAAE3* (*Oryza sativa AAE3*) on rice blast resistance by decreasing peroxidase activity [24]. However, oxalyl-CoA synthetase has not been studied in wild soybean.

In the present study, *GsAAE3* was cloned from *Glycine soja* (BW69), and its effects on tolerance to Cd and Al stresses were analyzed. The function of *GsAAE3* was identified and characterized by analyzing the kinetics and subcellular localization, along with performing a phenotypic analysis in transgenic *A. thaliana* lines and transgenic hairy roots of soybean overexpressing this protein. Based on our preliminary results, the oxalate-dependent GsAAE3 enzyme indirectly improves the Cd and Al tolerance of wild soybean by reducing the oxalate accumulation induced by both Cd and Al stresses.

## 2. Results

### 2.1. Oxalate Accumulation Induced by Cd and Al Contributes to Inhibit Root Growth in Wild Soybean

**Figure 1:**
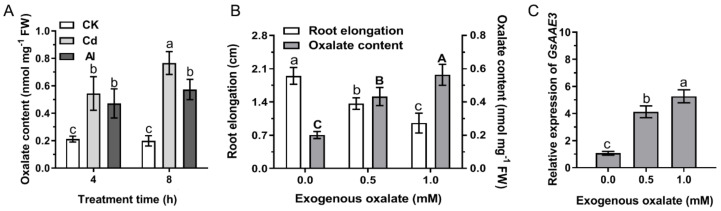
The effect of Cd and Al stresses and exogenous oxalate on wild soybean (BW69) root tips. (**A**) Cd and Al stresses induced oxalate accumulation. The seedlings were exposed to nutrient solution containing 0 or 30 μM CdCl_2_ or AlCl_3_ for 4 or 8 h. (**B**) The effect of exogenous oxalate on wild soybean root elongation and oxalate content. The seedlings were exposed to 0, 0.5, 1.0 mM sodium oxalate for 24 h. Root elongation was measured with a ruler before and after treatment (*n* = 16). After treatment, the root tips (0–2 cm) were ground into fine powder with liquid nitrogen and then extracted with distilled water for oxalate content analysis (*n* = 3). The lowercase letters mean statistical significance of comparisons of root elongation data, and the uppercase letters mean statistical significance of comparisons of oxalate content data. (**C**) Correlation between oxalate content and *GsAAE3* expression (*n* = 3). The expression of *GsAAE3* was determined by qRT-PCR. All data are presented as means ± SD. Different letters indicate statistically significant difference, using one-way ANOVA and Duncan’s test (*p* ≤ 0.05).

The oxalate content in wild soybean root tip was measured in response to Cd and Al stresses to confirm whether oxalate accumulated in wild soybean root tip during Cd and Al stresses and whether the accumulation of oxalate inhibited root growth. A significant accumulation of oxalate was observed in roots exposed to Cd and Al compared to the control groups. Furthermore, a greater increase in the oxalate content was observed at 8 h than at 4 h. No significant changes in the oxalate content were observed in the absence of Cd and Al (Figure 1A). On the other hand, the root elongation was dose-dependently inhibited by exogenous oxalate; conversely, the oxalate content in root tip increased continuously, consistent with the effect of oxalate accumulation induced by Cd and Al (Figure 1B). Based on these results, we postulated that oxalate accumulation induced by Cd and Al contributes to inhibiting root growth in wild soybean. In addition, we also measured the oxalate content in wild soybean root tips in response to Cu stress, and a significant accumulation of oxalate was observed in roots exposed to Cu at both 4 and 8 h compared to the control groups (Figure S4B), similar to the results of Cd and Al treatments. Based on the BW69 (*Glycine soja*) root tip Al stress-responsive genes expression profiles (unpublished), we found a gene, *GsAAE3*, whose expression was upregulated, induced by Al (Table S1), and it was annotated on NCBI as an oxalate-CoA ligase. Therefore, we speculated that *GsAAE3* expression was related to the reduction in oxalate accumulation. Interestingly, a quantitative real-time PCR experiment showed that *GsAAE3* expression is positively correlated with the oxalate content in roots (Figure 1C).

### 2.2. Isolation and Bioinformatics Analysis of the GsAAE3 Gene

**Figure 2:**
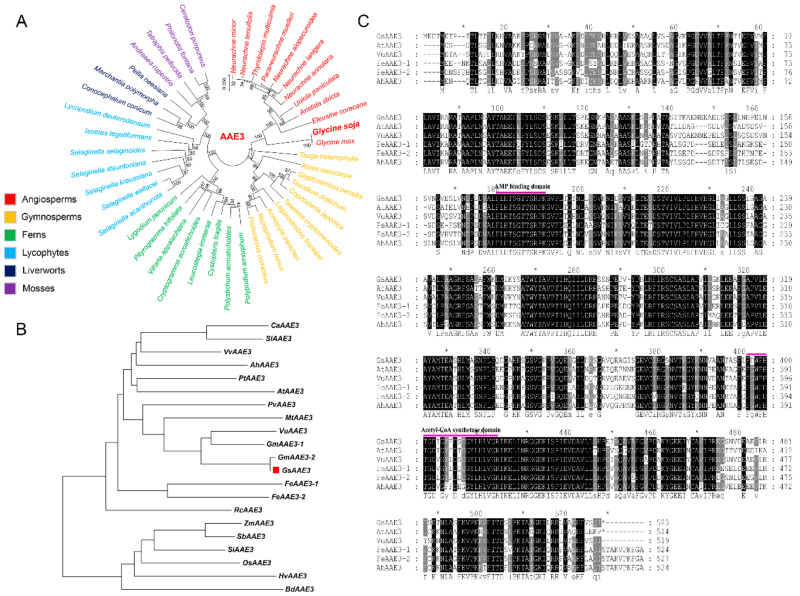
Phylogenetic analysis and multiple sequence alignment. (**A**) Phylogenetic trees of AAE3 proteins in representative species of major lineage of plants (sequences of representative species can be found in https://db.cngb.org/onekp/, accessed on 18 November 2020). Clades are indicated by different colors. (**B**) Phylogenetic analysis of AAE3 proteins in higher plants. All the available amino acid sequences and the accession numbers of AAE3 proteins were obtained from the NCBI databases (https://www.ncbi.nlm.nih.gov/, accessed on 20 October 2020). The AAE3 proteins including *Capsicum annuum* (CaAAE3; NP_001311686), *Solanum lycopersicum* (SlAAE3; XP_004234395), *Vitis vinifera* (VvAAE3; XP_002267459.1), *Amaranthus hypochondriacus* (AhAAE3), *Populus trichocarpa* (PtAAE3; XP_002322473), *Arabidopsis thaliana* (AtAAE3; AT3G48990), *Phaseolus vulgaris* (PvAAE3; XP_007143422), *Medicago truncatula* (MtAAE3; XP_003599555), *Vigna umbellate* (VuAAE3; KX354978), *Glycine max* (GmAAE3-1; XP_003534000.1, GmAAE3-2; XP_014619651.1), *Glycine soja* (GsAAE3; BW679327.1), *Fagopyrum esculentum* (FeAAE3-1 and FeAAE3-2), *Ricinus communis* (RcAAE3; XP_0002509782), *Zea mays* (ZmAAE3; AEY64280), *Sorghum bicolor* (SbAAE3; KXG27467), *Setaria italic* (SiAAE3; XP_004960018), *Oryza sativa* (OsAAE3; Os04g0683700), *Hordeum vulgare* (HvAAE3; BAK00674), and *Brachypodium distachyon* (BdAAE3; XP_003579506). (**C**) Multiple sequence alignment of AAE3 proteins from *Glycine soja* (GsAAE3), *Vigna umbellate* (VuAAE3), *Arabidopsis thaliana* (AtAAE3), *Medicago truncatula* (MtAAE3), *Amaranthus hypochondriacus* (AhAAE3), and *Fagopyrum esculentum* (FeAAE3-1 and FeAAE3-2). The conserved AMP binding domain and acetyl-CoA synthetase domain are indicated.

The full-length *GsAAE3* sequence was isolated from the root tip of BW69 (GenBank accession number: MN399820). The coding sequence (CDS) of *GsAAE3* is 1572 bp (Figure S1, Data S1A), encoding a protein of 523 amino acids with an isoelectric point of 6.16. Moreover, we utilized bioinformatics methods to investigate the evolution of GsAAE3 in plants. Analysis using the OneKP database (https://db.cngb.org/onekp/, accessed on 18 November 2020) of the 1000 plant species [25,26], we found that AAE3 was present in angiosperms and gymnosperms, even in some early-diverging plant lineages, such as ferns, lycophytes, liverworts, and mosses (Figure 2A). To further analyze the AAE3 in higher plants, the phylogenetic relationship was performed after comparing GsAAE3 with the amino sequences of twenty-one other known plant AAE3 members. As shown in Figure 2B, GsAAE3 and GmAAE3-2 from *Glycine max* are divided into a group, and GsAAE3 was more closely related to the AAEs of dicotyledonous plants than to those of monocots. Besides, a blast search was performed to determine the similarities between the target sequence and the sequences identified in the NCBI nonredundant protein sequence database. The results showed that the predicted GsAAE3 protein exhibits 77.4% similarity and 76% identity with AtAEE3 [20]. Additionally, GsAAE3 contains an (adenosine monophosphate) AMP binding domain and acetyl-CoA synthetase domain, similar to five other homologs, suggesting that these proteins may have similar functions (Figure 2C).

### 2.3. Analysis of the GsAAE3 Expression Pattern

**Figure 3:**
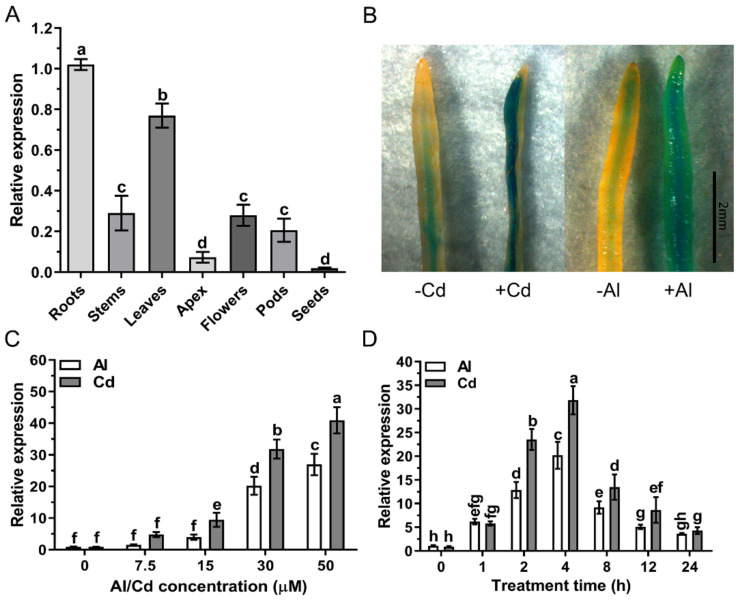
Expression pattern analysis of *GsAAE3*. (**A**) Tissue expression pattern of *GsAAE3*. The samples of whole roots, stems, leaves, apex, flowers, pods, and seeds were collected when the plants were already in seed-filling stage. (**B**) (β-glucuronidase) GUS staining in *GsAAE3* promoter-*GUS* transgenic hairy roots. (**C**) Dose-dependent expression of *GsAAE3* in wild soybean root tips (0–2 cm). The seedlings were cultured in the nutrient solution added with 0, 7.5, 15, 30, or 50 μM CdCl_2_ or AlCl_3_ for 4 h. (**D**) Time-dependent expression of *GsAAE3* in wild soybean root tips (0–2 cm). The seedlings were cultured in the nutrient solution added with 30 μM CdCl_2_ or AlCl_3_ for 0, 1, 2, 4, 8, 12, or 24 h. The expression of *GsAAE3* was determined by qRT-PCR. Three independent biological replicates were performed, and the data are presented as the means ± SD. Different letters indicate statistically significant difference, using one-way ANOVA and Duncan’s test (*p* ≤ 0.05).

Quantitative real-time PCR was performed to determine the relative expression of *GsAAE3*. *GsAAE3* was expressed in all plant organs, with the highest expression in roots and lowest in seeds (Figure 3A). Moreover, (β-glucuronidase) GUS staining in *GsAAE3* promoter-*GUS* transgenic soybean hairy roots indicated that GUS activity was observed in the vascular cylinder of root tip in the presence of Cd and Al stresses. Cd and Al stresses resulted in the increase in GUS activity in the hairy roots and it is stronger in the roots treated with Cd than that treated with Al (Figure 3B). In a dose-response experiment, *GsAAE3* relative expression increased as the external Cd and Al concentrations increased in the range of 0 to 50 μM (Figure 3C). In addition, in a time course experiment, the expression of *GsAAE3* was increased by 31.83- and 20.21-fold after 4 h of Cd and Al treatments, respectively. However, by the 8th hour of treatment with the same metals, the relative expression of *GsAAE3* had already decreased gradually from the peak levels (Figure 3D). Furthermore, we also analyzed the expression of *GsAAE3* with the treatment of Cu, and the result showed that, compared with the control group, Cu induced the upregulation of *GsAAE3* (Figure S4A), next only to Cd and Al.

### 2.4. GsAAE3 Encodes an Oxalyl-CoA Synthetase

**Figure 4:**
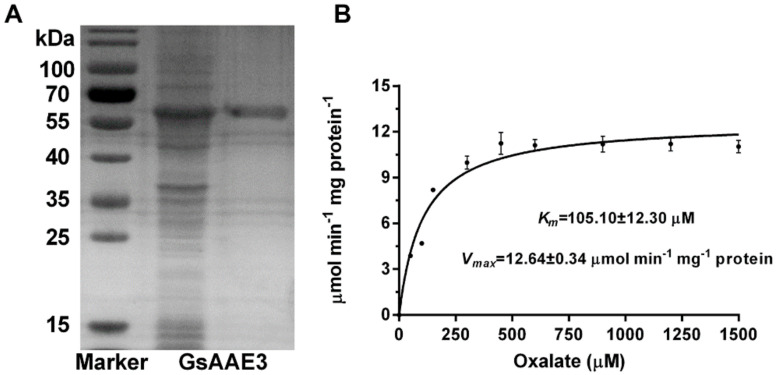
Biochemical analysis of *GsAAE3*. (**A**) SDS-PAGE gel of Ni-NTA purified recombinant protein (right), unpurified recombinant (middle), and molecular weight markers (left). (**B**) Kinetic analysis of *GsAAE3* using a range of oxalate concentrations. *K_m_* and *V_max_* were determined from non-linear regression to the Michaelis–Menten kinetics for concentrations up to 1500 μM oxalate. Data are means ± SD of three independent biological replicates.

The full-length cDNA of *GsAAE3* was obtained from *Glycine soja* and expressed as a His fusion protein in *E. coli* BL21 using the pET28a vector. Following purification using nickel affinity chromatography, the recombinant protein was estimated to be >90% pure, based on the electrophoresis pattern on the SDS-PAGE gel, and the protein weighed 56 kDa (Figure 4A). A kinetic analysis of GsAAE3 was conducted using a range of oxalate concentrations. At saturating concentrations of CoA (0.5 mM) and ATP (5 mM), the enzyme displayed Michaelis–Menten kinetics for oxalate concentrations up to 450 μM. Using these data, a *V_max_* of 12.64 ± 0.34 μmol min^−1^ mg^−1^ protein and a *K_m_* of 105.10 ± 12.30 μM was calculated (Figure 4B). The data are similar to that found for AtAAE3 and VuAAE3 and the differences are small for both *K_m_* and *V_max_* [14,20]. Based on these results, GsAAE3 also functions as an oxalyl-CoA synthetase.

### 2.5. Subcellular Localization of GsAAE3

**Figure 5:**
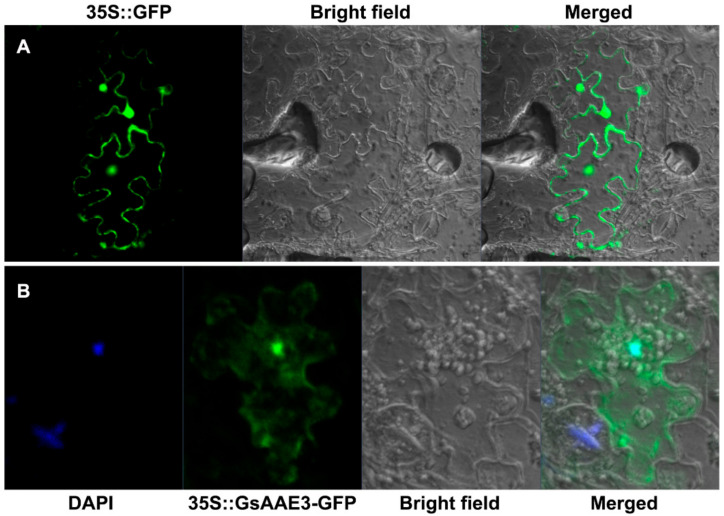
Subcellular localization of GsAAE3. (**A**,**B**) Green fluorescent protein (GFP) fluorescence images of tobacco leaves lower epidermal cells transiently expressing 35S::GFP and 35S::GsAAE3-GFP. DAPI (4′, 6-Diamidino-2-Phenylindole), a cell nucleus-specific fluorescence dye, was used to stain cell nucleus.

The WoLF PSORT program [27] (https://www.genscript.com/wolf-psort.html?src=leftbar, accessed on 17 November 2020) was used to predict the subcellular localization of GsAAE3 and the result showed that cytoplasm: seven, chloroplast: three, nucleus: two, vacuole: one (no specific subcellular localization). To further confirm the subcellular localization of *GsAAE3*, the coding sequence of *GsAAE3* was fused to the N-terminal side of green fluorescent protein (GFP) driven by the *Cauliflower mosaic virus* 35S promoter in pCAMBIA1302 to verify this prediction. We transiently expressed the 35S::GsAAE3-GFP fusion protein in tobacco leaves to observe the localization. Microscopic observation showed that the GFP signal was evenly distributed in the cell when the control vector (35S::GFP) was transiently expressed in tobacco leaves (Figure 5A). Nevertheless, the fusion protein 35S::GsAAE3-GFP was expressed in cytoplasm and nucleus in the tobacco lowered epidermal cells (Figure 5B), indicating that GsAAE3 has no specific subcellular localization.

### 2.6. Effects of GsAAE3 Overexpression in A. thaliana on the Tolerances to Cd and Al

**Figure 6:**
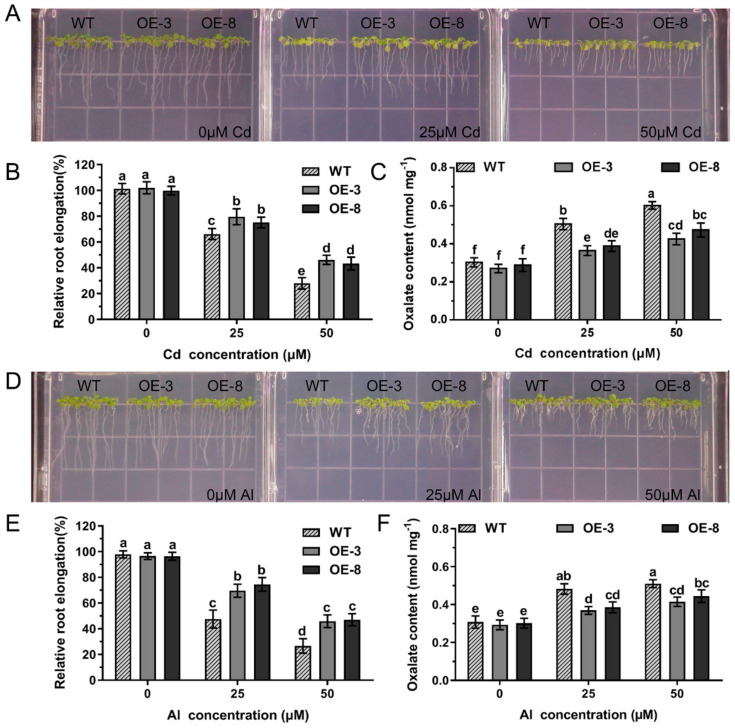
Overexpression of *GsAAE3* in *A. thaliana*. (**A**,**D**) Cd and Al tolerances phenotypes of overexpressing *GsAAE3* in transgenic *A. thaliana* lines. (**B**,**E**) Statistical analysis of relative root elongation. (**C**,**F**) The effect of Cd and Al stresses on oxalate content in wild-type and transgenic *A. thaliana* lines. Four days after vernalization, the T_3_ transgenic seeds (OE3 and OE8) and the wild-type (columbia-0) were cultivated on the Cd concentration gradients agar medium added with 0, 25, and 50 μM CdCl_2_ or Al concentration gradients agar medium added with 0, 25, and 50 μM AlCl_3_. After 7 days in culture, the images of the phenotypes of the *GsAAE3* transgenic lines were recorded for statistical analysis. The root elongation was measured using Image J software (*n* = 3). After treatment, the roots of *A. thaliana* were ground into fine powder with liquid nitrogen and then extracted with distilled water for oxalate content analysis (*n* = 3). All data are presented as means ± SD. Different letters indicate statistically significant difference, using one-way ANOVA and Duncan’s test (*p* ≤ 0.05).

Transgenic *A. thaliana* lines overexpressing *GsAAE3* were obtained to further confirm the role of *GsAAE3* in regulating plant responses to Cd and Al stresses. Ten transgenic *A. thaliana* lines overexpressing *GsAAE3* presented different expression levels (Figure S2). Two *GsAAE3*-overexpressing transgenic *A. thaliana* lines (OE3 and OE8) with relatively high expression levels were selected for subsequent phenotypic experiments examining Cd and Al responses. As shown in Figure 6A,D, neither the wild-type (WT) nor transgenic plants showed a difference in the relative root elongation in the absence of Cd and Al. However, in the presence of Cd and Al, the relative root elongation of *GsAAE3* transgenic lines was significantly greater than WT lines (Figure 6B,E). Thus, the overexpression of *GsAAE3* increases Cd and Al tolerances in *A. thaliana*. Moreover, we determined the oxalate content of transgenic and WT lines to further study whether the increased tolerance of Cd and Al in *GsAAE3* transgenic *A. thaliana* lines was related to oxalate accumulation. The results showed that the oxalate content was noticeably increased in transgenic and WT lines in the presence of Cd and Al, compared to 0 μM Cd and Al. Importantly, the oxalate content of the WT lines was significantly higher than that of the *GsAAE3* transgenic lines after treatments with Cd and Al (Figure 6C,F). These results illustrated that Cd and Al treatments increased oxalate accumulation in *A. thaliana*, while the *GsAAE3*-overexpressing lines exhibit reduced oxalate accumulation, thus increasing their tolerances to Cd and Al.

### 2.7. Effects of GsAAE3 Overexpression in Soybean Hairy Roots on the Tolerances to Cd and Al

**Figure 7:**
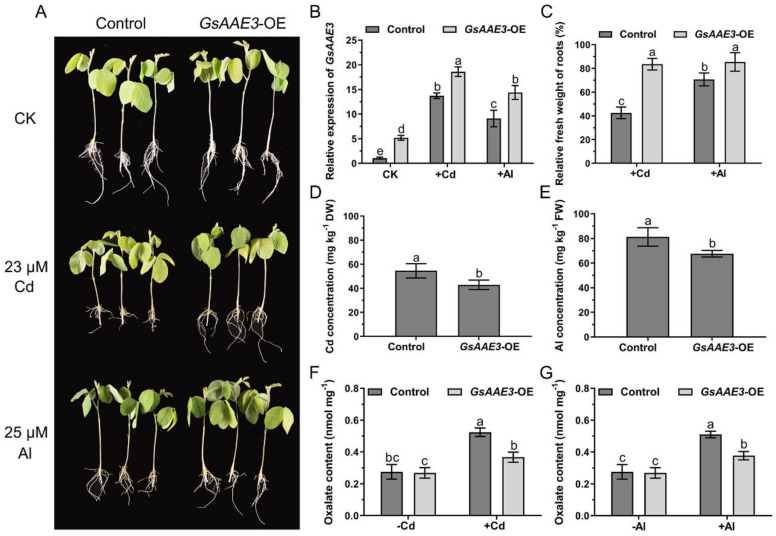
Performance of *GsAAE3* transgenic hairy roots under Cd and Al stresses. (**A**) Cd and Al tolerance phenotypes of overexpressing *GsAAE3* in soybean hairy roots. (**B**) Relative expression of *GsAAE3* in transgenic and control hairy roots under Cd and Al stresses and blank. (**C**) Relative fresh weight of hairy roots in transgenic and control plants under Cd and Al stresses. (**D**,**E**) The effect of Cd and Al stresses on Cd and Al concentrations in control and transgenic soybean hairy roots. (**F**,**G**) The effect of Cd and Al stresses on oxalate content in control and transgenic soybean hairy roots. The expression of *GsAAE3* was determined by qRT-PCR. The consistent growth positive transgenic and control hairy roots were chosen to transfer into nutrient solution supplemented with 23 μM CdCl_2_ or 25 μM AlCl_3_ or blank for 4 days. After treatment, the images of the phenotypes were recorded. The fresh weight and the concentrations of Cd and Al in transgenic and control hairy roots were measured. In addition, the soybean hairy roots were ground into fine powder with liquid nitrogen and then extracted with distilled water for oxalate content analysis. Three independent biological replicates were performed, and the data are presented as the means ± SD. Different letters indicate statistically significant difference, using one-way ANOVA and Duncan’s test (*p* ≤ 0.05).

To further investigate the role of *GsAAE3* under Cd and Al stresses, the vector (pTF101.1-*GsAAE3*) was introduced into soybean hairy roots for overexpression (*GsAAE3*-OE) analysis. The average expression level of *GsAAE3* in *GsAAE3*-OE hairy roots was 5.1-fold higher than that in the control hairy roots (Figure S3). Under Cd and Al stresses, we found that the expression of *GsAAE3* in both transgenic and control soybean hairy roots were upregulated with varying degrees (Figure 7B). In addition, as shown in Figure 7A, the control hairy roots exhibited greater sensitivity to Cd and Al stresses than soybean hairy roots overexpressing *GsAAE3*. The relative fresh weight of the control hairy roots was significantly lighter than the *GsAAE3* transgenic hairy roots in the presence of Cd and Al (Figure 7C). Moreover, Cd and Al were presented at significantly lower concentrations in the hairy roots of the transgenic lines than in the controls (Figure 7D,E). In general, the overexpression of *GsAAE3* increases Cd and Al tolerances in soybean hairy roots. Meanwhile, we also determined the oxalate content of transgenic and control hairy roots to further study whether the increased tolerance of Cd and Al in *GsAAE3* transgenic hairy roots was related to oxalate accumulation. The results are shown in Figure 7F,G; Cd and Al treatments increased oxalate content in soybean hairy roots and the oxalate content of the control hairy roots was higher than that of the *GsAAE3* transgenic hairy roots after treatments with Cd and Al. Based on these results, it can be concluded that *GsAAE3* enhances Cd and Al tolerances in soybean hairy roots by reducing oxalate accumulation.

## 3. Discussion

In the present study, we cloned *GsAAE3* from *Glycine soja* and showed that it encodes an oxalyl-CoA synthetase. Evidence supporting this finding is based on the degradation of oxalate catalyzed by the GsAAE3 protein and the observation that the GsAAE3 protein exhibits 77.4% similarity and 76% identity with AtAEE3 [20], which has been identified in *A. thaliana* as a protein encoded by *AAE3*. Among the other proteins with high homology to GsAAE3, VuAAE3 is most similar to GsAAE3 in function, which converts oxalate to oxalyl-CoA, thereby preventing oxalate toxicity induced by Al stress [14]. Analogously, AtAAE3 and MtAAE3 with high homology to GsAAE3 also exhibit certain resistance to environmental stress, defending against phytopathogens that secrete oxalate [20,22]. These results indicate that *GsAAE3* plays an important role in biotic and abiotic stress. Acyl-activating enzymes (AAEs), which are present in a wide range of living organisms, participate in an immense variety of anabolic and catabolic pathways [28]. A number of *AAE3* genes in different plant species are induced by pathogens, such as *Xanthomonas campestris* [23], rice blast fungus [24], and the oxalate-producing fungal pathogen [20,22], confirming that *AAE3* plays a significant role in plant resistance to biotic stress. Recently, Peng et al. reported that the *OsAAE3*-encoded protein functions as an oxalyl-CoA synthetase and regulates the resistance to Al toxicity [29]. Another *AAE3* member in rice bean (*VuAAE3*) also play an important role in tolerance to Al stress [14]. However, further research is needed to investigate the role of *AAE3* in the plant’s resistance to abiotic stress.

In the research of abiotic stress, the toxicities of Cd and Al has always been a limiting factor for the normal growth of plants [30,31]. In the preliminary experiment, *GsAAE3* relative expression was induced by Cd and Al stresses in the root apex of wild soybean (Figure 4B), and *GsAAE3* is the key gene in the CoA-dependent pathway of oxalate degradation. Therefore, we postulated that oxalate degradation is related to Cd and Al tolerances in plants. This speculation is supported by the evidence described below. Firstly, both Cd and Al stresses resulted in oxalate accumulation (Figure 1A). Secondly, the rapid accumulation of oxalate in root apices under Cd and Al stresses and the fact that *GsAAE3* relative expression was positively correlated with the oxalate content in roots (Figure 1C) suggested that oxalate might be a mediator that induces *GsAAE3* expression in response to Cd and Al stresses. Finally, the concentration of exogenous oxalate positively correlated with the oxalate content in roots and negatively correlated with the elongation of wild soybean roots (Figure 1B). Thus, the accumulation of oxalate is toxic to the roots of wild soybean. Oxalate occurs as a natural product in a wide range of plants and plays a crucial role in various biological processes [22]. In plants, oxalate functions in the regulation of Ca^2+^, ion balance, and osmotic pressure in cells and defends against plant pathogens, herbivores, and metal poisoning [7,32,33,34,35,36,37]. Although the biosynthesis and accumulation of oxalate plays a role in the normal growth of the plant, excessive accumulation of oxalate is harmful to plant growth and development [17,22]. Finally, in the presence of Cd and Al, the *GsAAE3* transgenic *A. thaliana* and soybean hairy roots exhibited an obvious decrease in oxalate accumulation and showed higher tolerances to Cd and Al (Figure 6 and Figure 7).

In the analysis of Al tolerance, our results are similar to of the findings from the study by Lou et al., who reported that *VuAAE3* plays a significant role in Al tolerance and oxalate accumulation contributes to Al-induced inhibition of root growth [14]; thus, the Al tolerance mechanisms regulated by *GsAAE3* may closely resemble *VuAAE3*. However, inconsistent results have been recently reported showing that down-regulation of *OsAAE3* increases Al tolerance in rice [29], which was associated with an increase in oxalate accumulation. The explanation for these two opposite results may be that different plants have different levels of sensitivity to oxalate. In the oxalate accumulator plants, sufficient oxalate would be secreted to chelate metal ions when plants were subjected to metal stress, while at this point, excessive oxalate is also a stress to the oxalate non-accumulator plants (such as soybean). Hence, these oxalate non-accumulator plants would control the oxalate content in vivo to indirectly alleviates the damages bring from metals. Further research is needed to determine how plant sensitivity to oxalate affects the direct or indirect response of oxalate to stress. On the other hand, the results of the analysis of Cd tolerance are similar to the results of Al tolerance; thus, we propose that an analogous mechanism underlying the tolerances of *GsAAE3* to Cd and Al exists. Generally, *GsAAE3* improves the tolerances to Cd and Al by decreasing oxalate accumulation induced by Cd and Al. In a previous study of Cd and Al tolerances, many genes related to Cd and Al were extensively analyzed. For instance, the Al-induced secretion of organic acids in the root tips of many Al-resistant plants is considered an important mechanism of Al tolerance in plants. As early as the 1990s, aluminum was reported to induce the secretion of citrate, malate, and oxalate in snapbean, wheat, and buckwheat [8,38,39]. With the development of plant research on Al tolerance, an increasing number of Al tolerance genes that encode organic acid transporters have been identified in various plants [40,41,42,43,44]. These genes belong to two gene families: multidrug and toxic compound extrusion (MATE) and Al-activated malate transporter (ALMT). MATEs mainly mediate the secretion of citrate, while ALMTs mainly mediate the secretion of malate [43,45]. In soybean, numerous reports have also confirmed that the aluminum tolerance of soybean is related to the secretion of citrate and malate [46,47,48], while few reports have described the mechanism by which oxalate regulates Al tolerance. Soybean is an oxalate non-accumulating plant, and thus, the role of oxalate in the mechanism of Al tolerance in soybean is novel and worth studying. In the study of Cd tolerance, several key genes have been shown to regulate the absorption and accumulation of Cd in plants, including members of the ABC transporter family, ZIP family, HMA family, and other families [49,50,51]. However, the role of *GsAAE3* in Cd tolerance in soybean has not been investigated. Unlike the general tolerance mechanisms, which act directly on metal ions, *GsAAE3* was shown to improve the Cd and Al tolerances in wild soybean is through an indirect route by degrading oxalate in the present study.

To the best of our knowledge, we believe that this report is the first to describe the function of *AAE3* in the tolerances of wild soybean to Cd and Al. Through this study, we suspected that the function of *AAE3* in the response to heavy metal toxicity may be conserved in oxalate non-accumulating plants, but this hypothesis must be validated in a prospective study. To date, the mechanisms by which oxalate accumulates in oxalate non-accumulating plants remain unknown. Three main biosynthetic pathways of oxalate in plants have been proposed: the glyoxylate pathway, ascorbic acid pathway, and oxaloacetic acid pathway [35,52,53,54,55]. Although the pathway mediating oxalate production in *GsAAE3*-regulated tolerance mechanisms remains unclear, oxalate accumulation has been shown to play a role in the responses to biotic and abiotic stresses [5,12]. In the oxalate-accumulating plant, buckwheat, harmful metal ions induce oxalate accumulation or secretion to alleviate toxicity [8], but the non-accumulating plants, wild soybean and rice bean, mainly deal with harmful metal ions by secreting citrate that forms a stable complex with toxic metal ions in the rhizosphere [47,56]. Therefore, compared with the oxalate accumulating plant buckwheat, the identification of the oxalate metabolic pathway in the oxalate non-accumulating plant soybean is particularly important.

Oxalate is a common constituent of plants, and it accumulates primarily as soluble oxalate, insoluble calcium oxalate, or a combination of these two forms [2]. The statistics of the content of water-soluble oxalates and total oxalates in some plants were carried out by Liber et al. [2], and they found that ontogenetic stage, light conditions, temperature, and nutrient status will all influence oxalate accumulation and proportion. In addition, studies have shown that calcium oxalate crystals are ubiquitous in higher plants and account for up to 90% of the total plant calcium [57]. Calcium oxalate crystal deposition can occur within the vacuoles of cells or associated with the cell wall [3], which are important for chelation detoxification of harmful metals in plants.

Currently, heavy metal pollution and Al toxicity are the focus issues worldwide, because both heavy metals and Al are toxic to plants [58,59]. More importantly, heavy metal accumulation in the edible parts of crops potentially threatens human health [60,61]. In recent years, with the development of molecular research, an increasing number of plant resistance genes has been shown to respond to harmful metal stress through proteins translation [62,63], and these molecular mechanisms of plant tolerance have been gradually explored and applied in breeding different plant varieties [64,65].

In conclusion, in the present study, we cloned and characterized *GsAAE3*, which increased Cd and Al tolerances in wild soybean. Moreover, our results provided a foundation for further investigations of the functions of AAE3 in the responses to other harmful metal stresses in oxalate non-accumulating plants.

## 4. Materials and Methods

### 4.1. Plant Material and Treatments

The seeds of BW69 (Al-resistant) lines of *Glycine soja* were gently cut from the seed coat on the back of the hilum with a knife and germinated in sand. After germination, the seedlings were pre-cultured in 0.5 mM CaCl_2_ (pH = 4.5). After two days, the seedlings displaying consistent growth were transferred into a nutrient solution (pH = 4.5) described by Lou et al. [14] with the following composition: 200 μM CaSO_4_, 200 μM CaCl_2_, 100 μM MgSO_4_, 400 μM KNO_3_, 300 μM NH_4_NO_3_, 5 μM NaH_2_PO_4_, 3 μM H_3_BO_3_, 0.5 μM MnCl_2_, 0.4 μM ZnSO_4_, 0.2 μM CuSO_4_, 10 μM Fe-EDTA, and 1 μM (NH_4_)_6_Mo_7_O_24_. After two days of culture, the seedlings were transferred into a nutrient solution (pH = 4.5), containing different treatments. For the time dependence experiment, seedlings were cultured in the nutrient solution added with 30 μM CdCl_2_ or AlCl_3_ for 0, 1, 2, 4, 8, 12, or 24 h. For the Cd and Al concentrations dependence experiment, seedlings were cultured in the nutrient solution added with 0, 7.5, 15, 30, or 50 μM CdCl_2_ or AlCl_3_ for 4 h. In addition, for the tissue dependence experiment, the seeds were planted in potted soil and the roots, stems, leaves, apex, flowers, pods, and seeds were collected when the plants were in the seed-filling stage. For the exogenous oxalate experiment, the seedlings were exposed to 0, 0.5, or 1.0 mM sodium oxalate for 24 h. Root tissues of the seedlings (0–2 cm) and the other tissue samples were lyophilized and stored at −80 °C until use. The hydroponic system referenced Cai et al. [66]; all nutrient solutions were refreshed daily, and all experiments were performed in an environmentally controlled culture room with 12 h light at 200 μmol photons/m^2^/s, 70% humidity, and 22–25 °C.

### 4.2. Quantitative Real-Time PCR (qRT-PCR) and Gene Expression Assay

Total RNA was isolated from the roots and the other tissue samples using the Plant Total RNA Purification Kit (TR02-150, GeneMarkbio, Taiwan). Extracted RNA was purified from the contaminating DNA and reverse transcribed using a PrimeScript^TM^ RT reagent Kit with gDNA Eraser (RR047A, TaKaRa, Shiga, Japan). For qRT-PCR, solutions were mixed with SYBR Premix Ex Taq^TM^ II (RR820A, TaKaRa, Shiga, Japan) and analyzed using the CFX96 Real-Time System (Bio-Rad, Hercules, CA, USA). The data were normalized to the reference gene *GmActin6* (AK285830.1) or *AtActin2* (*At3g18780*). The qRT-PCR data were evaluated using the 2^−ΔΔCt^ method [67]. The experiment was performed with three independent biological replicates. The CDS of *GsAAE3* was amplified from the cDNA of *GsAAE3* that had been reverse-transcribed from the total RNA using Super-Fidelity DNA polymerase (P505, Vazyme, Nanjing, China). The corresponding primers used in the present study are listed in Table S2.

### 4.3. Bioinformatics Analysis and Statistical Analysis

The blast tool on the National Center for Biotechnology Information (NCBI) website (https://blast.ncbi.nlm.nih.gov, accessed on 20 October 2020) was used to search for homologous proteins to the amino acid sequence of *GsAAE3*. The early-diverging plant AAE3 protein sequences were chosen using the One Thousand Plant Transcriptome database (https://db.cngb.org/onekp/, accessed on 18 November 2020) according to Leebens-Mack et al. [26]. DNAMAN software was used to construct the multiple sequence alignment and MEGA7.0 software was used to generate homology tree. For the statistical analysis, SPSS Statistics (v. 22) and GraphPad Prism 6 software were used to analyze and process the data. Significant differences were evaluated using one-way ANOVA and Duncan’s test at *p* ≤ 0.05.

### 4.4. Subcellular Localization

The subcellular localization of the proteins was analyzed using a previously described method [68], with slight modifications. The full-length CDS of *GsAAE3* was cloned into pCAMBIA1302 to generate the *35S::GsAAE3::GFP* recombinant plasmid. After sequencing, the constructs were transformed into *Agrobacterium tumefaciens* GV3101. The cultures of viral protein p19 and transformed *Agrobacterium* GV3101 were resuspended in an infiltration buffer (10 mM MES-KOH, 10 mM MgCl_2_ and 100 μM acetosyringone) after centrifugation and then mixed at a 1:1 volumetric ratio. After 3–4 h incubation, the mixed bacteria solution was injected into the leaves of 3-week-old *Nicotiana tabacum* L. plants with a syringe. The GFP signal from the subepidermal cells of tobacco was observed after 60 h under a confocal laser scanning microscope (Carl Zeiss, Jena, Germany). The corresponding primers used in the present study are listed in Table S2.

### 4.5. A. thaliana Transformation and Phenotypic Analysis

The CDS of *GsAAE3* was cloned into the expression vector pTF101.1, and protein expression is driven by the CaMV 35S promoter. Then, the pTF101.1-*GsAAE3* was introduced into the *Agrobacterium tumefaciens* strain GV3101 before transformation into the *A. thaliana* plants using the floral-dip method [69]. Positive transgenic lines were selected with 10 mg/L Basta and continuously screened for three generations. The corresponding primers used in the present study are listed in Table S2.

For the phenotypic analysis, transgenic seeds (T_3_), and wild-type seeds (columbia-0) were cultivated on agar medium containing Cd concentration gradients [70] or agar medium containing Al concentration gradients [71]. Next, the seeds were incubated at 4 °C in the dark for 4 days before they were transferred to a 22 °C culture room and cultured vertically for 7 days with 16 h light at 200 μmol photons/m^2^/s, 50% humidity.

### 4.6. Soybean Hairy Root Transformation and Phenotypic Analysis

The pTF101.1-*GsAAE3* and the empty vector were introduced into soybean hairy roots via transformation with the *Agrobacterium rhizogenes* strain K599 to further elucidate the functions of *GsAAE3* [72]. The transgenic hairy roots were cultured in the nutrient solution according to Lou et al. [14]. After 2 days of growth on nutrient solution, the transgenic hairy roots displaying consistent growth were transferred into renewed nutrient solution (pH 4.5) supplemented with 23 μM CdCl_2_, 25 μM AlCl_3_, or a blank control for four days [14,73]. Subsequently, the soybean hairy roots were harvested and used to measure the fresh weight, oxalate content, and Cd and Al contents. The used for the determination of Cd and Al contents by inductively coupled plasma–atomic emission spectrometry (ICP-AES).

### 4.7. GUS Staining

For further elucidating the expression pattern of *GsAAE3*, total DNA was isolated from the roots of *Glycine soja* (BW69) using the New Plant Genome Extraction Kit (DP320, TIANGEN, Beijing, China). The DNA sequence located 1819 bp upstream of the initiation site in *GsAAE3* (Data S1B) was cloned into pCAMBIA3301 to generate the *GsAAE3* promoter-*GUS* fusion plasmid under the control of CaMV 35S promoter. The *GsAAE3* promoter-*GUS* fusion plasmid was introduced into soybean hairy roots via transformation with the *Agrobacterium rhizogenes* strain K599 [72]. After the culture in the nutrient solution added with 23 μM CdCl_2_ or 25 μM AlCl_3_ for 4 h, the soybean hairy roots were stained for GUS. The GUS activity of transformed roots was detected using the GUS Stain Kit (RTU4032, Real-Times). The corresponding primers used in the present study are listed in Table S2.

### 4.8. Root Growth Analysis

For the analysis of soybean root growth, the root length was measured with a ruler, and the root fresh weight was measured with the electronic balance. Image J software was used to measure the root length of *A. thaliana*. The root elongation was computed as (root length after treatments—root length before treatments). The relative root elongation was computed as (root length with treatments/root average length without treatments) × 100%. The relative fresh weight of roots was computed as (root fresh weight with treatments/root fresh weight without treatments) × 100%.

### 4.9. Purification of the Recombinant Protein and Kinetic Analysis

The coding sequence of *GsAAE3* was cloned into the pET28a vector and transferred into the *Escherichia coli* BL21 (DE3) strain (Baldwin et al., 2001). The expression of the recombinant protein was induced by 1 mM isopropyl-β-d-thiogalactopyranoside (IPTG) at 30 °C for 4 h. After sonication and centrifugation, the filtered fusion protein was purified with Ni-NTA His-Bind Resin (PAN001, Toscience), and the purified protein was detected by separation on a 12% SDS-PAGE gel. The corresponding primers used in the present study are listed in Table S2.

The enzymatic activities of purified recombinant protein were determined using sodium oxalate as the substrate with a coupled enzyme assay [20,74]. The assay was conducted by the mixing 5 μg of the purified protein, a range of concentrations of the substrate, and the buffered reaction mixture containing 0.1 M Tris-HCl (pH 8.0) or 0.1 M NaPO_4_ (pH 8.0), 2 mM DTT, 5 mM ATP, 10 mM MgCl_2_, 0.5 mM CoA, 0.4 mM NADH, 1 mM phosphoenol-pyruvate, and 10 units each of myokinase, pyruvate kinase, and lactate dehydrogenase together in a total volume of 800 μL. The reaction was monitored by measuring the oxidation of NADH at 340 nm in the presence of different oxalate concentrations [14] using an ultraviolet visible spectrophotometer (Shimadzu Co., Kyoto, Japan). The experiment was performed with three independent biological replicates.

### 4.10. Oxalate Measurement

The plant materials were ground into a fine powder with liquid nitrogen and then extracted with distilled water. The extract was centrifuged at 12,000 rpm for 10 min. The supernatant solution was collected and pass first through a cation exchange column and then through an anion-exchange column. HCl (1 M) was used as eluent to collect oxalate retained in the anion-exchange resin [14]. The oxalate content was determined using ICS-3000 ion chromatography. The mobile phase of oxalate was 30 mM NaOH, and the flow rate was 0.6 mL min^−1^ [14]. Three independent biological replicates were tested in each experiment for oxalate measurement.

## Supplementary Materials

Supplementary Materials can be found at https://www.mdpi.com/1422-0067/21/22/8869/s1.

## Abbreviations

Cd	Cadmium
Al	Aluminum
AAE3	ACYL-ACTIVATING ENZYME 3
GFP	Green fluorescence protein
qRT-PCR	Real-time quantitative polymerase chain reaction
CDS	Coding sequence
WT	Wild-type
DTT	DL-Dithiothreitol
ATP	Adenosine triphosphate
