# Supplementary material for: Wild Soybean Oxalyl-CoA Synthetase Degrades Oxalate and Affects the Tolerance to Cadmium and Aluminum Stresses
Source: Int J Mol Sci. 2020 Nov 23;21(22):8869. doi: 10.3390/ijms21228869 (PMC7700444; doi:10.3390/ijms21228869)
Supplement: Supplementary file 1 [file ijms-21-08869-s001.pdf]

Supplementary Material

1 Supplementary Data

Data S1A

CDS sequencing information of *GsAAE3* (GenBank accession number: MN399820)

ATGAAAGACACAATGGAAACTCCTACGACACTCACCACCTTGCTCCGCCACGTCGC
CGCCAAATTCCCCTCCCGCCGTGCCATCTCCGTCGCTGCCAAATTCGACCTCACCCA
CTCCCGCCTCCACCGCCTGGTCGAATCCGCCGCCGCTCAGTTAGTCTCCGCCGGCGT
CAAACCCGGCGACGTCGTCGCCCTCACCTTCCCCAACACCATCGAGTTTGTAGTCAT
GTTCCTAGCCGTCATTCGGGCTCGAGCCACAGCCGCGCCGTTAAACTCGGCTTACAC
AGCGGAAGAGTTCGAGTTTTACTTATCCGACTCGGAGTCCAAGCTCCTCCTAACATC
ACCAGAAGGAAACAAGCCTGCTCAAGCCGCGGCTTCAAAGCTCAGCATCCCCCAC
GCCACGGCTTCGATTACCAAAGCCGAGAACGAAGAAGCGGAGCTGAGTCTCTCATT
ATTAAACCACCCCGAGTTGAATTCCGTTAACTCAGTTGAGTCACTCGTTAACGACCC
TGATGACGTGGCACTTTTCCTTCACACCTCTGGCACCACGAGCCGTCCCAAGGGAGT
GCCGCTGACTCAGTACAACCTTCTCTCTTCGGTGAAGAACATCGACTCAGTGTACCG
ACTCACTGAGTCCGACTCGACGGTGATTGTTCTTCCTCTCTTCCACGTGCACGGGTTA
ATCGCCGGATTGCTGAGCTCCCTCGGCGCCGGAGCTGCGGTGGCGCTTCCGGCGGC
GGGGAGGTTCTCCGCCTCGGCGTTTTGGAAGGACATGATTAAATACAGCGCCACAT
GGTACACCGCGGTTCCCACCATACACCAGATCATACTTGATCGCCACTCCAGCAAC
CCCGAACCGGTTTACCCGCGGCTCCGGTTCATCCGAAGCTGCAGCGCCTCGCTCGC
ACCGGTTATACTCGGTAAACTCGAGGAGGCTTTCGGTGCACCGGTTTTAGAGGCCTA
TGCGATGACGGAAGCGTCGCATTTGATGGCTTCGAACCCTTTGCCACAAGATGGGG
CCCACAAGTCCGGGTCGGTTGGGAAACCCGTGGGTCAGGAAATGGGCATTTTAGAC
GAATCGGGTCGGGTCCAAGAGGCTGGTATCAGTGGAGAGGTTTGCATTCGGGGATC
CAATGTTACGAAAGGATATAAAAATAACGTGGCAGCAAATACGGCGTCGTTTCTGT
TCGATTGGTTTCATACTGGTGATATTGGATACTTTGATTCTGATGGGTATTTGCATCT
CGTGGGTCGGATCAAGGAACTCATCAACCGAGGAGGTGAAAAGATATCACCAATA
GAAGTGGATGCTGTTCTTCTTTCACATCCAGAAATTGCTCAAGCAGTTGCTTTTGGA
GTGCCTGATGCCAAGTATGGAGAGGAGATATATTGTGCTGTCATCCCAAGAGAAGG
ATCAAACGTTGATGAGGCAGAGGTGCTAAGATTTAGCAAGACCAATCTTGCATCCT
TCAAAGTACCAAAAAAGGTCTTCATTACTGATTCTTTACCCAAAACTGCCACAGGC
AAGATTTTGCGCCGTCTTGTGGCAGAACACTTTGTCTCTCAAATTTGA

Data S1B

Promoter sequencing information of *GsAAE3* (GenBank accession number: MN684205)

GTCTTGCATACAATCACTCACATTATGACAAGTCTTTAAAATTTTAAAATAACATAT
AAAATATTAAATTAAAGTCATGTTACTGTTAATTCTTTCCCCCTTGCAAGCCATAAC
CTTTACCAATCCTCCTCAAATGTCGTGGCCCACACCTCCAATCTCCAGAGCCCCACC
ATAACCATCAATCTCAGTTTCTAAAGTGGAATCAATGCTACGATTTTGACACAAGTA
AAAAAATAATCAATGTTATGCTTTAAAACAATTATTTAATTAGGTCTGTTCACAAAC
TATTAAAGATGAAATTTGTTGAATAAAGGAATAATTAGAAGACAAACACGCATCCA
TATAAAACACTTTTTTTTGCCGCAATTATTTTAATTAAAGAAAAATCCCCTTGCAATC
TGTTTGGCATGGTACCTCAACATGTAAACTCATAATAAGGGAACACCCTACTCAAT
AAGCAGCTTCACCAAAGACCCTCCTTTTAGAAGTCCCATTTGTCGACGACGATCTCT
AAGGAGAGGTGATGCATGCTTTGGGAAGAGGTGTTGATGGTGATCTTGAAGGAGGT
GACCGTGGCGGCTTGGGGAGGTGTAGGCTATTTGAAGAAAGGAGGGTTGGGTTGAG
AGAGAAGATGAGTTTACATTCAAGTGGATCCTTTTAATTTTGTTCATATATTTGTTTT
TTATTTTTAGATTTTATGAGTCGTCACTTTTTAAGTGGTTGTGTACAAAAATGACGTG
TTGCGTGTGTATATACAAGACTTCCTCGTAATTTCTATTGTTGAAAAACATGAAACA
ATAATAGTCACAATGGATAACTAGGGTTTTTCAATTAACTTTTTGTTAAACACAAGT
AGTGATCACCCGTCAGTGTTACTTGAGTTATTTTTTTTTTCTTTTTTTACAAAATATAC
TTGAGTTGGTCACTTGATTTTTTTTTGTTAGACACAACTAGTTATCCGCATCATAAAC
AGTAAAGATCAACCTAAAATTCAAATTCTAAATCACTTATTAAAAAACATAATTCA
TTATATTTTTGTGAATCTTTGTTAGTTGTATTCTGATTTTATTATTTTCAATATGTTTTA
ATTAATAAAAAGTATATATATATATATATATATATATATATATATATATATTTGTATTAC
TAGTCCTCCCGATTCTGATTATCTTTCTCGAAAACAAACCTTCCCCAGATAAAGAGT
TAGTGGAAAAATTTAATAAATGCATGTAGGGCAAAAAAAAGAGGAAAACTCCGAG
ACTCTCCAACTCCAAATTTCTCGTCAAAGTCTCCAATAGTCACATCAAGAAATAAA
AAAGTATAGTTAGTGAATAAATTGAAAAACTAATTTACTTTAATAACTCAAATCGA
TCTACGGTCAATTTCCTCTTTGATTAATAAATATATTAATCAATTTAATCCATTACAT
TATAAAAATAATTTAGTTATTAACTTAGTACTAATTACTAACCGCTAGTTTTATTTAT
AATTTCGTAGATTAATTTAATTCTTGTTTTTATTTTATTTAAGAATTTATAATAAATAA
AAATAATTCTATTAAAGTAGAAGCTGGGTGCTGGCCAGACAGATTTGACTACGCGG
CGTGTGCCAATGCCATTGTACACGAACCATCCCACTCTTCTCCATTCCACGAACCCA
ACTTTGCCTTCTTTTTCATTCCCAGATACCACCTTCCCTCTATCCCTATATATATATAT
TAACACAACACAAATTCACAACTCATTCACTTCACAATTCATCAACATCTAACACC
ACACACACGCAACACGCTTCAGCCACTATTCTTCAAGGAAAAACAAA

2 Supplementary Figures and Tables

2.1 Supplementary Figures

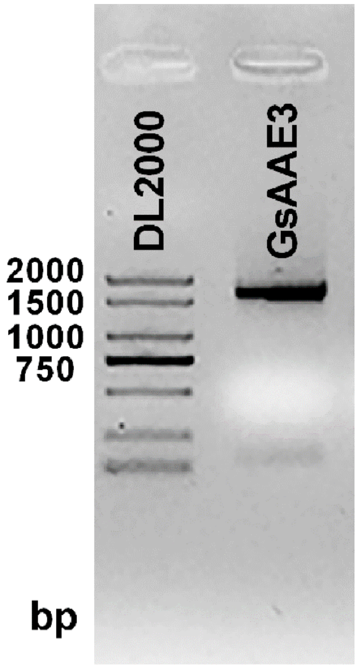

Supplementary Figure S1. The *GsAAE3* CDS fragment was examined by agarose gel electrophoresis.

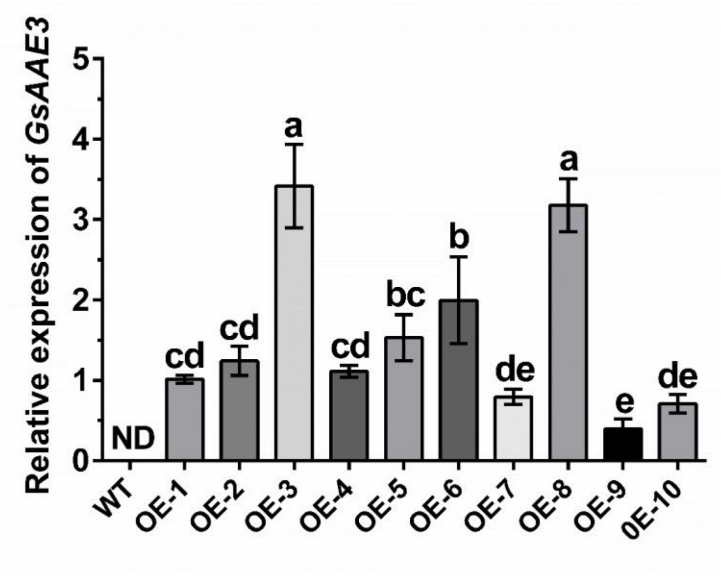

Supplementary Figure S2. Quantitative real-time PCR (qRT-PCR) analysis of overexpressing *GsAAE3 Arabidopsis* lines. ND, not detected. Data are means ± SD of three

biological replicates. Different letters indicate statistically significant difference, using one-way ANOVA and Duncan's test ($P \leq 0.05$).

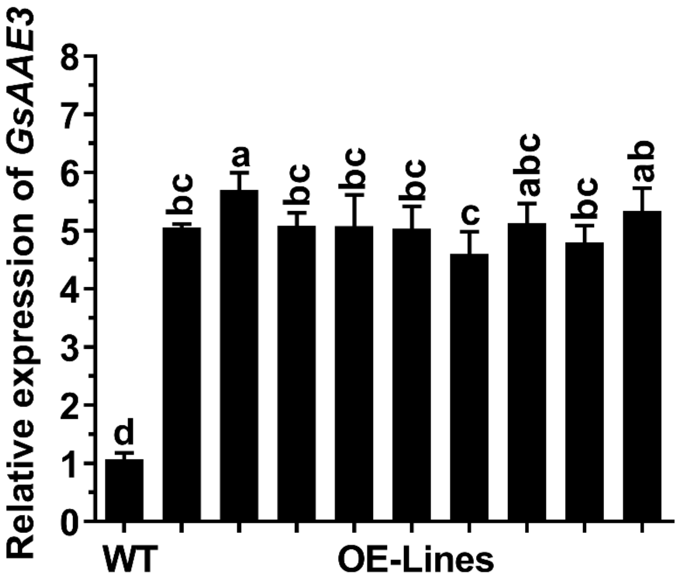

Supplementary Figure S3. Quantitative real-time PCR (qRT-PCR) analysis of overexpressing *GsAAE3* soybean hairy roots. Data are means ± SD of three biological replicates. Different letters indicate statistically significant difference, using one-way ANOVA and Duncan's test ($P \leq 0.05$).

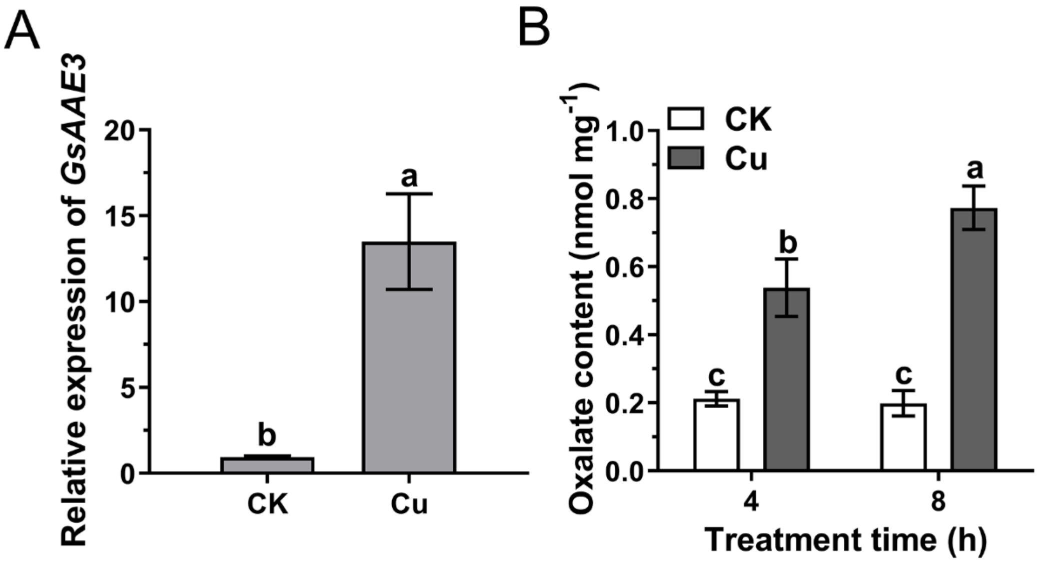

Supplementary Figure S4. (A) Relative expression of *GsAAE3* in wild soybean root tips (0-2 cm). The seedlings were cultured in nutrient solution as control (CK) or the same nutrient solution added with 30 μM $CuCl_2$ for 4 hours. (B) The effect of Cu stress on wild soybean root tip oxalate content. The seedlings were exposed to nutrient solution containing 0 or 30 μM $CuCl_2$ for 4 or 8 h. Data are means ± SD of three biological replicates. Different letters indicate statistically significant difference, using one-way ANOVA and Duncan's test ($P \leq 0.05$).

2.2 Supplementary Tables

Table S1 The data analysis of *GsAAE3* gene expression profiles from BW69 line of *Glycine soja*.

Gene	Gene ID	GenBank Acc	Description	Log2(Al/CK)
GsAAE3	100817853	BW679327.1	Oxalate--CoA ligase / Oxalyl-CoA synthetase	1.71

Table S2 List of primers.

Serial number	Primer name	Primer sequence (5'-3')	Purpose
1	*GsAAE3*-F	ACACGCTTCAGCCACTATTC	*GsAAE3* CDS cloning
2	*GsAAE3*-R	CTCTATTATTGTATCCTGTAGG	
3	GsAAE3-proF	GTCGCACTCCATTAGGCTATG	*GsAAE3* promoter cloning
4	GsAAE3-proR	AAACTCGAACTCTTCCGCTGT	
5	pTF101-*GsAAE3*-F	gagaacacgggggactctagaATGAAAGACACAATGGAAACTCC	*GsAAE3*-pTF101.1
6	pTF101-*GsAAE3*-R	cgatcggggaaattcgagctcTCAAATTTGAGAGACAAAGTGTTCTG	
7	p3301-*GsAAE3*-F	tatgaccatgattacgaattcGTCTTGCATACAATCACTCACATTATG	*GsAAE3*-pCAMBIA3301
8	p3301-*GsAAE3*-R	ttaccctcagatctaccatggTTTGTTTTTCCTTGAAGAATAGTGGC	
9	p1302-*GsAAE3*-F	tatgaccatgattacgaattcctATGAAAGACACAATGGAAACTCC	*GsAAE3*-pCAMBIA1302
10	p1302-*GsAAE3*-R	tactagtcagatctaccatggAATTTGAGAGACAAAGTGTTCTGCC	
11	pET28a-*GsAAE3*-F	cagcaaatgggtcgcggatccATGAAAGACACAATGGAAACTCC	*GsAAE*3-pET28a
12	pET28a-*GsAAE3*-R	gtggtggtggtggtgctcgagTAATTTGAGAGACAAAGTGTTCTGCC	
13	GmActin6F	GCACCACCGGAGAGAAAATA	qRT-PCR
14	GmActin6R	GTGCACAATTGATGGACCAG	
15	AtActin2F	GGTATTGTGCTGGATTCTGG	
16	AtActin2R	CGCTCTGCTGTTGTGGTGA	
17	q*GsAAE3*-F	TACGGCGTCGTTTCTGTT	
18	q*GsAAE3*-R	CTCGGTTGATGAGTTCCTTG	
19	35S-F	CCTTCGCAAGACCCTTCCTC	Molecular identification
20	GUS-R	GTTGGGGTTTCTACAGGACG	
